# Two-Dimensional Scattering Center Estimation for Radar Target Recognition Based on Multiple High-Resolution Range Profiles

**DOI:** 10.3390/s24216997

**Published:** 2024-10-30

**Authors:** Kang-In Lee, Jin-Hyeok Kim, Young-Seek Chung

**Affiliations:** 1 Defense Rapid Acquisition Technology Research Institute, Agency for Defense Development, Seoul 07062, Republic of Korea; leekangin@add.re.kr; 2 Group for Quantum Electricity & Magnetism Metrology, Korea Research Institute of Standards and Science, Daejeon 34113, Republic of Korea; jinhykim@kriss.re.kr; 3 Department of Electronic Convergence Engineering, Kwangwoon University, Seoul 01897, Republic of Korea

**Keywords:** high resolution range profile, microwave propagation, radar target recognition, scattering center estimation, target signature

## Abstract

A new estimation strategy on locations of two-dimensional target scattering centers for radar target recognition is developed by using multiple high-resolution range profiles (HRRPs). Based on the range information contained in multiple HRRPs obtained from various observation angles, the estimated target scattering centers can be successfully located at the intersection points of the lines passing through the multiple HRRP points. This geometry-based algorithm can significantly reduce the computational complexity while ensuring the ability to estimate the two-dimensional target scattering centers. The computational complexity is formulated and compared to that of the conventional methods based on the synthetic aperture radar (SAR) images and HRRP sequences. In order to verify the performance of the proposed algorithm, the numerical and experimental results for three different types of aircraft were compared to those from SAR images. At the end of this article, the estimated radar scattering centers are used as the target features for the conventional classifier machine to confirm its target classification performance.

## 1. Introduction

Target recognition technology has been used in the military field to improve the early assessment of potential threats, such as missiles and hostile aircraft, and to respond with appropriate means. Reliable target recognition capabilities in coastal surveillance and air traffic control systems can also monitor and confront intrusions of illegal immigrants, smugglers, and terrorists by land or sea [1]. In addition to the military field, target recognition algorithms that are implemented with radar systems to detect pedestrians or unexpected oncoming threats have become a breakthrough technology in the autonomous vehicle industry.

Target signatures commonly used for target recognition include wideband radar cross-section (RCS) frequency response, high-resolution range profile (HRRP), and synthetic aperture radar (SAR) images. Multiple wideband RCS frequency responses for different angles are generally used to recognize small targets compared to the system range resolution. Huang and Lee [2] proposed a target identification method using the RCS data for three different targets. In this method, the similarity between the unknown and known targets was defined to perform a statistical classification, where the dataset was based on the RCS data that are observed in directions of different elevation and azimuth angles.

If a range profile provides sufficient information to identify a target, either the HRRP or the SAR image can be used in the target recognition process. The HRRP is a one-dimensional representation of the target characteristics obtained by expressing the distribution of target scattering points projected onto the observation axis. Despite the low computational complexity, accurate target discrimination cannot be achieved due to the possibility that similar HRRPs can be obtained from different targets [3]. On the contrary, in the SAR images, which are expressed in a two-dimensional target scattering point distribution, the probability of having similar characteristics from different targets is relatively low, but higher computational complexity is required to obtain high-dimensional target characteristics [4].

In terms of using the HRRPs and SAR techniques for real-time recognition, the former, which can be obtained by performing the one-dimensional inverse Fourier transform of the received signals, has an advantage over the latter, which requires complex signal processing, such as two-dimensional Fourier transforms and back projection of the HRRPs obtained from multiple angles [5,6]. However, as Novak evaluated in [7], the classification results from two-dimensional algorithms using SAR images show better performance than those from one-dimensional HRRPs.

Over the past few decades, numerous studies have been conducted to secure both performance and computational efficiency, which can be divided into two main categories according to their approaches. Studies in the first category are focused on developing effective feature extraction methods as preprocessing techniques prior to classification. Numerous feature extraction methods have been introduced since the early 2000s, including principal component analysis (PCA) [8,9,10,11,12] and linear discriminant analysis (LDA) [13,14,15,16], and their common purpose was mainly to reduce redundancy and dimensionality in extracting a feature vector for a given target. Studies in the second category are focused on designing robust classifiers, such as the Bayesian decision-based classifier, pattern recognition-based classifier and neural network-based classifier [17,18]. With advances in computing and information technologies, object classification capabilities of machine learning (ML) methods, such as the support vector machine (SVM) [19,20] and the extreme learning machine (ELM) [21], have been widely utilized in the field of target recognition.

However, since both approaches are biased towards the processing of the acquired data, they cannot be freed from the inherent limitations of the target signature itself, which are mentioned in the fourth paragraph of this article. To mediate the trade-off between classification accuracy and computational load in target recognition, some studies on developing the estimation method of target scattering centers have been conducted since the early 2000s.

Kyung-Tae Kim and Hyo-Tae Kim [22] proposed a new method that estimates two-dimensional locations of target scattering centers using a set of one-dimensional down-range locations and associated amplitudes over several aspect angles. In this method, the one-dimensional total least squares estimation of signal parameters was applied via rotational invariance techniques, and an intersection point equation based on geometric techniques, such as projection and triangulation, was derived to provide input training data for multiple elastic modules (MEMs). As a result, the two-dimensional locations of target scattering centers were estimated using an iterative scheme for MEM network optimization. The authors showed better estimation accuracy and computational complexity compared to the well-known two-dimensional matrix enhancement and matrix pencil technique. However, it is also stated that the algorithm tends to rely on the initial guess of module center locations due to its iterative nature, and the computational complexity can be significantly increased when the number of aspect angles increases.

Jianxiong et al. [23] proposed a reconstruction method, which estimates three-dimensional scattering centers from multiple HRRPs without knowledge of aspect angles or relative radar-target movement based on the signal space estimation method. In this method, the singular value decomposition technique was used to estimate signal subspace, and the performance was evaluated by Cramer–Rao Lower Bounds of the reconstruction problem. However, the uniqueness of the solution in the algorithm could not be ensured due to an arbitrary rotation matrix between the reconstructed and real scattering center coordinates. To overcome the ambiguity in [23], Jun et al. [24] developed a parameter matrix based on the geometric projection of the scattering center on the line of radar sight in multi-aspect, which is unique for any rotational translation. The effectiveness of the method was investigated for a flat-bottomed cone target with five scattering points, and the authors emphasized the advantages in resolution, stability, data requirement, and computational complexity. Recently, Su et al. [25] pointed out the ambiguity in [23] and the degradation of performance with increasing numbers of scattering centers and pulses. In this article, the authors divided the spatial target scattering centers with micro-motion properties into two types: stable and sliding scattering centers. Based on the factorization method used [24], the coordinates of stable and sliding scattering centers were reconstructed in three-dimensional and two-dimensional space, respectively. However, this method still cannot ensure the uniqueness of the solution, and at least six aspect angles are required to perform the scattering center estimation.

Ji-Hoon Bae and Kyung-Tae Kim [26] proposed a compressive sensing (CS)-based two-dimensional scattering center extraction (SCE) technique to extract scattering centers from incomplete radar cross-section (RCS) data. Unlike traditional methods, this approach is designed to accurately extract scattering centers when some RCS data are missing. However, due to the nature of the compressive sensing technique used in the proposed method for estimating scattering centers, the method inherently has high computational complexity.

Jianxiong et al. [27] proposed a method for estimating a global 3D scattering center model using multiple HRRPs. In this method, the HRRPs obtained from various angles are visualized on an OTSM (One-Two/Three-Dimensional Scatter Map), and a Hough Transform is then applied to automatically convert the 1D projection positions into 3D scattering centers. In [28], the 3D scattering centers of the target obtained using the method from [27] were pre-acquired and then used to build a database for SAR image-based automatic target recognition.

In summary, the methods described in [27,28] involve observing the target model of interest from numerous aspect angles and investing significant computational resources to estimate the global 3D scattering center model before new observations and identification. This model is then used to build a database necessary for target recognition using SAR images.

However, the method proposed in this paper can acquire target signatures with less computational complexity than the SAR images in environments where moving targets are being tracked, serving as an alternative to the SAR images. Therefore, it differs in purpose from the methods in [27,28]. Additionally, due to the computational complexity of the dataset required in [27] and the Hough Transform, the proposed method has significantly lower computational requirements compared to [27].

There is a certain trade-off between the estimation accuracy and computational load, both proportional to the number of dimensions to be processed. Therefore, we propose a method for constructing target signatures that provides dimensional qualities similar to those of a SAR technique with greatly reduced computational load. In the proposed method, the geometric relationship between multiple HRRPs and the location of scattering centers was used to define an error function based on the least-squares method (LSM), and two-dimensional locations of target scattering centers were estimated to represent the points, such as edges, corners, and planes, where the RCSs were expected to be large. Compared to the previous methods in [22,23,24,25], the estimation performance of the algorithm is fully proportional to the quality of HRRPs obtained by the radar system, and only three aspect angles of HRRP are required to ensure the uniqueness of the result. In addition, the estimation process is applicable even in an environment in which the number of visible scattering centers varies depending on the observation angle. Due to the non-iterative nature based on the geometric approach, the computational complexity of the proposed algorithm can be significantly reduced.

The remainder of this article is organized as follows. In Section 2, the scattering center estimation method for a point target model is developed using multiple HRRPs and expanded to be applicable for an actual target model. The computational complexity of the method is compared to that of the conventional techniques. In Section 3, the numerical and experimental results of scattering center estimation are provided using measured HRRPs from three different types of aircraft and applied to the conventional classifier to confirm its target classification performance. Finally, conclusions are given in Section 4.

## 2. Scattering Center Estimation

In this section, a strategy for the two-dimensional estimation of radar target scattering centers is developed based on multiple HRRPs and their geometric relations. The multiple HRRPs are assumed to be obtained in the same manner as a spotlight-mode-SAR observation model that collects radar signals for various aspect angles by continuously steering the radar beam at a target on a rotating platform [29].

### 2.1. Point Target Model

**Figure 1:**
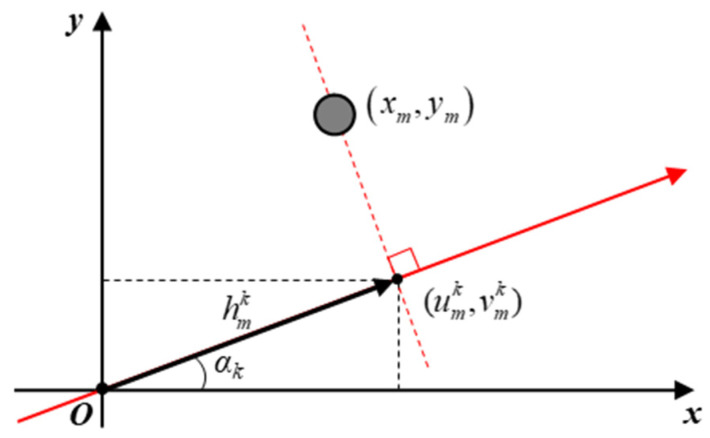
Concept of HRRP projection onto the observation axis.

The HRRP contains the range information of target scattering centers projected onto the observation axis. A basic geometric concept of the HRRP projection is shown in Figure 1. Since $\left(u_{m}^{k},v_{m}^{k}\right)$ is the point at which the scattering center $\left(x_{m},y_{m}\right)$ is projected onto the observation axis, the range profile can be expressed as

(1) $$h_{m}^{k}=x_{m}\cos\left(\alpha_{k}\right)+y_{m}\sin\left(\alpha_{k}\right)$$

where $h_{m}^{k}$ is the profile which contains range information of the *m*-th scattering center with the *k*-th observation angle *α_k_*. The range profile in (1) can be obtained from the peak location in the HRRP, which is the inverse discrete Fourier transform of the received signal in the frequency domain. Therefore, the resulting magnitude of $h_{m}^{k}$ in (1) is equal to the distance of a point $\left(u_{m}^{k},v_{m}^{k}\right)$ from the origin.

Using the obtained range profile with known value of $\alpha_{k}$, the projected point $\left(u_{m}^{k},v_{m}^{k}\right)$ can be defined as

(2) $$\left(u_{m}^{k},v_{m}^{k}\right)=\left(h_{m}^{k}\cos\left(\alpha_{k}\right),h_{m}^{k}\sin\left(\alpha_{k}\right)\right)$$

The imaginary line passing through (2) and perpendicular to the observation axis can be formulated as

(3) $$y=-\frac{\cos\left(\alpha_{k}\right)}{\sin\left(\alpha_{k}\right)}\left(x-u_{m}^{k}\right)+v_{m}^{k}\;$$

Substituting (2) in (3), another expression for the range profile $h_{m}^{k}$ can be obtained by

(4) $$h_{m}^{k}=x\cos\left(\alpha_{k}\right)+y\sin\left(\alpha_{k}\right)$$

By the definition of (4), the same range profile can be obtained from any point on the imaginary line passing through the projected point and perpendicular to the observation axis. Clearly, the real scattering center $\left(x_{m},y_{m}\right)$ is located on the line defined by (4), which yields (1), but it is impossible to inversely specify the exact location from $h_{m}^{k}$ alone. Therefore, in the case shown in Figure 1, more range profiles are needed to decide the exact coordinate of the scattering center. With the help of several range profiles obtained from different observation angles, a set of simultaneous equations from (4) can be derived, which can be finally solved for the unknown coordinates of scattering centers.

Once the multiple HRRPs are obtained for *M* scattering centers with *K* observation angles, the total dataset can be expressed in matrix form as

(5) h=xP

where

$$h=\left[\begin{array}{cccc} h_{1}^{1} & h_{1}^{2} & \cdots & h_{1}^{K}\\ h_{2}^{1} & h_{2}^{2} & \cdots & h_{2}^{K}\\ \vdots & \vdots & \ddots & \vdots \\ h_{M}^{1} & h_{M}^{2} & \cdots & h_{M}^{K} \end{array}\right],x=\left[\begin{array}{cc} x_{1} & y_{1}\\ x_{2} & y_{2}\\ \vdots & \vdots \\ x_{M} & y_{M} \end{array}\right],P=\left[\begin{array}{cccc} \cos\left(\alpha_{1}\right) & \cos\left(\alpha_{2}\right) & \cdots & \cos\left(\alpha_{K}\right)\\ \sin\left(\alpha_{1}\right) & \sin\left(\alpha_{2}\right) & \cdots & \sin\left(\alpha_{K}\right) \end{array}\right].$$

The rows and columns of **h** represent the obtained HRRPs for different angles and scattering centers, respectively. The **x** matrix contains two-dimensional location of scattering centers, and **P** is the projection matrix, whose column vectors are ${\left(\cos(\alpha_{k}),\sin(\alpha_{k})\right)}^{T}$.

Considering *K* observations, the simultaneous equations for the *m*-th scattering center can be expressed in the matrix form as

(6) $$h_{m}=x_{m}P$$

where $h_{m}$ is the 1-by-*K* size row vector whose elements are $\left[h_{m}^{1},h_{m}^{2},\cdots ,h_{m}^{K}\right]$ and $x_{m}$ is the row vector that contains the location of scattering center $\left(x_{m},y_{m}\right)$.

Based on the LSM, the location of *m*-th scattering center can be estimated by matrix inversion of (6) as follows:

(7) $$x_{m}=h_{m}P^{†}$$

where (∙)^†^ is the pseudo-inverse operation. As stated above, the two-dimensional location of a single scattering center is able to be specified by finding the solution of simultaneous equations in (7), which can be described as the intersection point of the vertical lines from two different HRRPs. However, there are two issues to be simultaneously considered under the general circumstances: authenticity of the solution and the matching problem.

**Figure 2:**
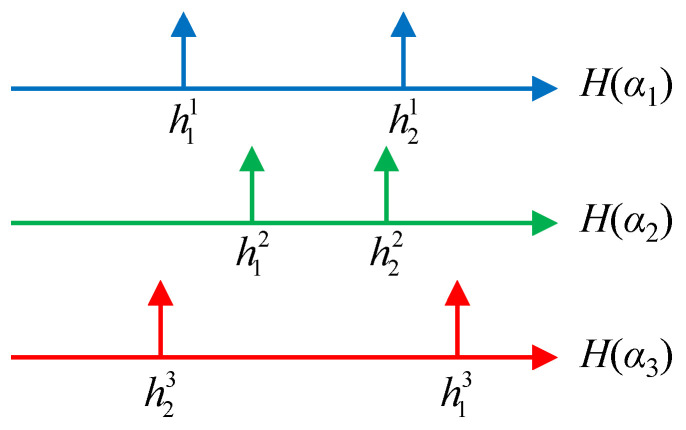
Target information shown as peaks on HRRP axes for three observation angles.

As shown in Figure 2, the targets are observed in the form of peaks on the HRRP axes, but they appear in different order depending on the observation angle, so the target scattering center index *m* for each peak is actually unknown. If the components of matrix **h** in (5) are ‘mismatched’ to the order of actual targets, the unique solution cannot be obtained.

**Figure 3:**
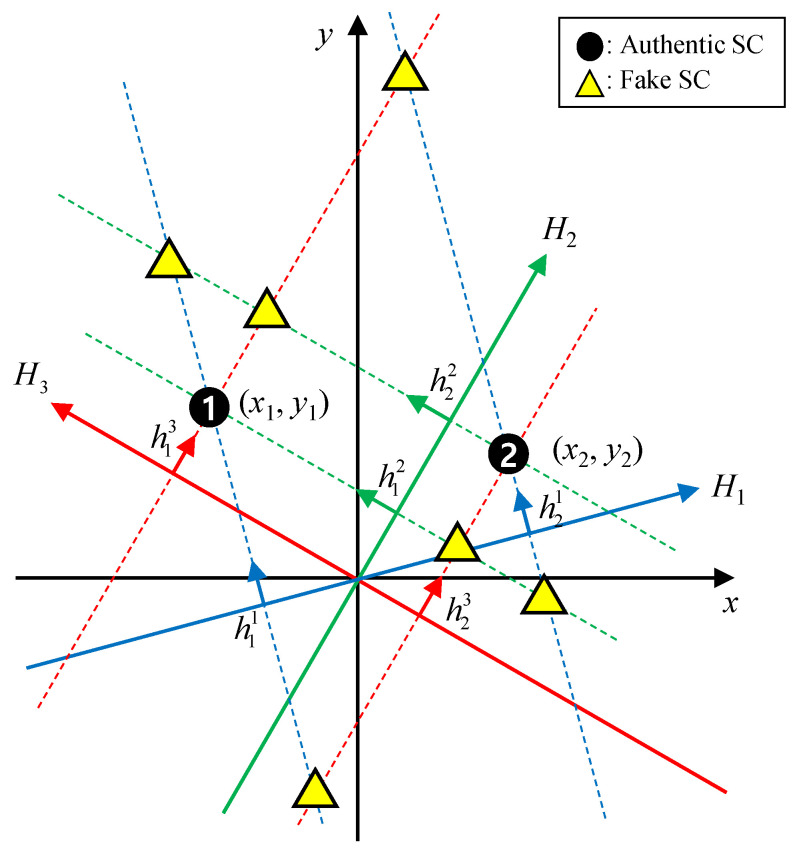
HRRPs for three observation angles when two scattering centers exist.

Figure 3 shows an example of two scattering centers observed from three different angles. The dotted lines represent the vertical lines passing through the projected scattering centers on each HRRPs, derived as in (4). In Figure 3, two observation axes of $H_{1}$ and $H_{2}$ generate four intersections of $(h_{1}^{1},h_{1}^{2}),$ $(h_{1}^{1},h_{2}^{2}),$$(h_{2}^{1},h_{1}^{2}),$ and $\left(h_{2}^{1},h_{2}^{2}\right)$. Of these four points, only two intersections, created by $\left(h_{1}^{1},h_{1}^{2}\right)$ and $\left(h_{2}^{1},h_{2}^{2}\right)$, are the authentic scattering centers, while the other two are not. Therefore, to sort out the real solution, one more range profile $H_{3}$ is needed. As shown in Figure 3, the authentic scattering centers are located only when the three vertical lines intersect, which means the unique solution of (7) can be obtained when K=3.

However, even with the three range profiles, the three vertical lines may not intersect, as the components of **h** may still be mismatched, resulting in the obtaining of an incorrect solution of (7). In this case, the LSM places the solution point at the center of gravity of the triangle formed by the three lines. Therefore, to overcome the matching problem, an authentication algorithm is proposed based on the error defined by the geometric property of all possible combinations of HRRP peaks.

The number of combinations can be calculated as

(8) $$Q=\prod_{k=1}^{K}q_{k}$$

where $q_{k}$ is the number of peaks on *k*-th observation axis. In most cases, $q_{k}$ is equal to the number of scattering centers *M*, but it may be less depending on the location of the scattering centers and observation angle. 

To sort out the matched combinations, each of them is used as a HRRP vector in (7) to estimate the scattering center $x^{\prime }$. If the incorrect combination was used, the unique solution cannot be obtained, resulting in $x^{\prime }$ being located at the center of gravity of the triangle formed by the mismatched combination of HRRP vertical lines. Therefore, an error function can be defined based on the geometric relationship between each combination of HRRP peaks and the HRRP recalculated from the point estimated by the corresponding combination. The error function is defined as

(9) $$\varepsilon_{i}={\left\|{h^{\prime }}_{i}-{x^{\prime }}_{i}P\right\|}_{2},\;i=1,\;2,\ldots ,Q$$

where ${x^{\prime }}_{i}$ denotes the estimated location of the scattering center from $h_{i}$, the *i*-th combination of HRRP peaks, and ‖∙‖_2_ denotes the *L*_2_ norm.

**Figure 4:**
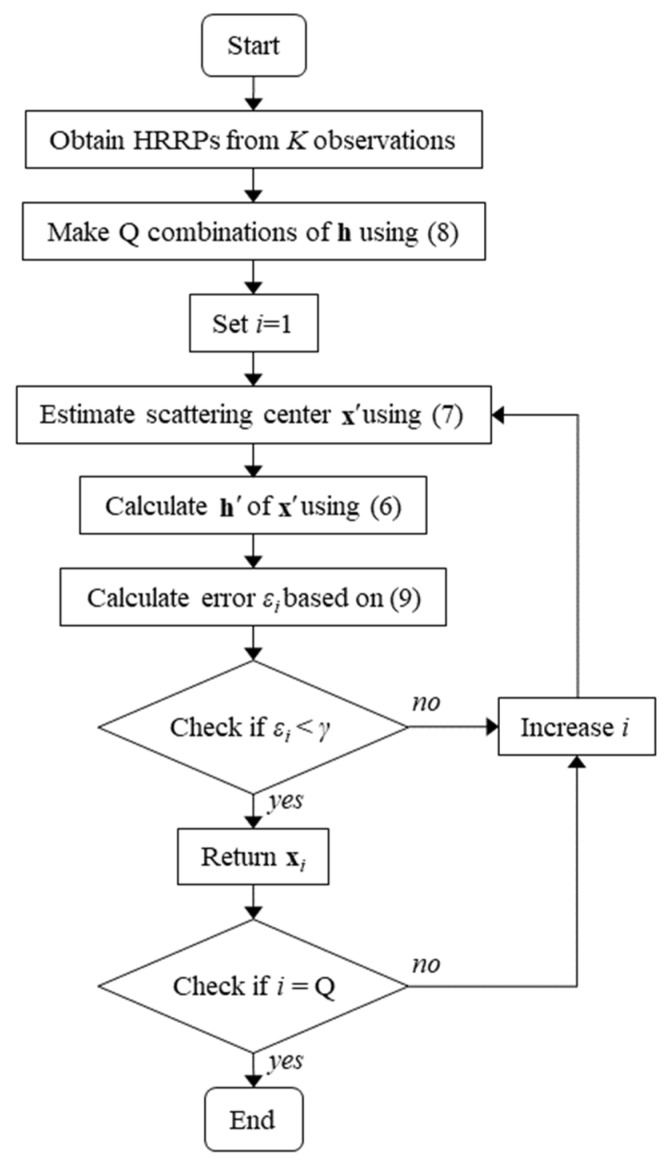
Flowchart of the proposed algorithm for two-dimensional radar target scattering center estimation.

Theoretically, the number of zeros out of the *Q* errors obtained by (9) should be equal to the number of authentic scattering centers. However, in practice, even the authentic scattering centers cannot have exactly zero errors due to the limitations of the radar measurement system. Therefore, we should introduce a threshold γ to the errors to determine the authenticity. The threshold γ must be less than or equal to ΔR/2 to ensure that the target exists within the same range-bin as the measured HRRP, where ΔR is the range resolution of the measurement system. The flowchart of the proposed algorithm is shown in Figure 4. The overall procedure can be summarized as follows:

Step 1: Obtain HRRPs from *K* observations
Step 2: Make *Q* combinations of HRRPs by considering the number of peaks on the *k*-th observation axis using Equation (8)
Step 3: Estimate the scattering centers using Equation (7) for each of the Q combinations of HRRPs. Note that the scattering centers estimated in this step include fake SCs.
Step 4: Calculate the HRRPs for all estimated scattering centers using the *K* observations and Equation (6).
Step 5: Calculate the error between the combinations of HRRPs obtained in Step 2 and the HRRPs estimated through Step 3 and Step 4 using Equation (9).
Step 6: If the calculated error in Step 5 is smaller than the threshold, the scattering centers are classified as authentic.

### 2.2. Actual Target Model

The point target model assumes that all scattering centers can be observed by the radar. That is, each HRRP contains the range information for all scattering centers. However, in the actual target model, there are cases where a specific region is not visible depending on the observation angle. Fortunately, multiple HRRPs obtained from adjacent angles have the information of the scattering centers within the same area, so the proposed algorithm can be applied to estimate the entire scattering centers on the target.

**Figure 5:**
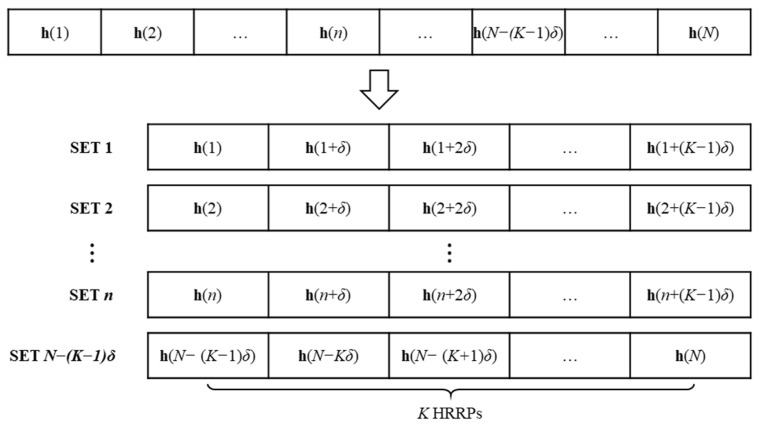
Generation of *N* − (*K* − 1)*δ* sub-datasets by choosing *K* neighboring HRRPs out of the entire HRRP dataset of size *N*.

Figure 5 shows the concept of choosing *K* neighboring HRRPs out of the entire HRRP dataset of size *N* to estimate all scattering centers around the target. In this figure, **h**(*n*) is the *n*-th observed HRRP, *δ* is the interval of each HRRP selection, and [h(n), h(n+δ), …, h(n+(K−1)δ)] are the *K* HRRPs used for the estimation procedure. Therefore, the proposed algorithm derived for the point target model can be applied to each of the N−δ(K−1) neighboring sets. The resulting scattering points estimated through the above process are used for further processing to find the dominant scattering centers located on an object’s surface that are likely to be observed from multiple viewpoints, such as edges, corners, and planes.

Based on the property of the dominant scattering centers, we introduce an exposure factor that defines the frequency at which the scattering centers estimated using each of the neighboring HRRPs are observed in the entire dataset. The HRRPs for all observation angles of the *i*-th scattering center can be calculated as follows:

(10) $${h^{\prime }}_{i}\left(n\right)={x^{\prime }}_{i}p\left(n\right),\;n=1,2,\ldots ,N$$

where ${x^{\prime }}_{i}\equiv [x_{i},y_{i}]$ and **p**(*n*) ≡ [cos(*α_n_*), sin(*α_n_*)]***^T^***.

The error between the estimated HRRP ${h^{\prime }}_{i}(n)$ and the measured HRRP can be expressed as follows:

(11) $$\beta_{i}\left(n\right)=\min\left(\left|h\left(n\right)-{h^{\prime }}_{i}\left(n\right)\right|\right)$$

where min(∙) stands for the minimum value of the vector elements. To verify the existence of the scattering center at a specific observation angle, the error obtained in (11) is applied to

(12) $$D_{i}\left(n\right)=\;\left\{\begin{array}{c} 1,\;\beta_{i}\left(n\right)<\gamma \\ 0,\;\beta_{i}\left(n\right)>\gamma \end{array}\right.$$

where γ is the threshold used for the point target model.

Using (12), the exposure factor of the *i*-th scattering center $\rho_{i}$ is given by

(13) $$\rho_{i}=\frac{1}{N}\sum_{n=1}^{N}D_{i}\left(n\right)$$

The locations of the dominant scattering centers of the actual target model can be finally estimated by comparing (13) to the exposure threshold *γ_ρ_*.

The overall performance of the proposed algorithm depends on the values of *K* and *δ*. In the theoretical environment, K=3 is a sufficient condition for two-dimensional estimation, but if the range resolution of the radar system is considered, the performance can be clearly improved in proportion to *K*. However, as the value of *K* increases, the computation complexity also increases as well, which is discussed in the following section. Smaller values of *δ* allow more scatter centers to be estimated, but the pseudo-inverse matrix inversion of **P** in (2) becomes more singular because the column vectors of the matrix **P***^T^***P** are not orthogonal. Therefore, the parameter *δ* should be determined considering the condition number of **P [30]**.

The overall estimation process for the actual target model can be summarized as follows:

Step 1: Estimate the location of the scattering centers using (7) and (9) from observed HRRP data.
Step 2: Estimate HRRP ${\hat{h}}_{i}\left(n\right)$ using (10).
Step 3: Calculate the error between the estimated HRRP and measured HRRP *β_i_*(*n*) using (11).
Step 4: Compare (11) to threshold *γ* to determine whether the *i*-th scattering center is exposed from the observation angles.
Step 5: Process all estimated scattering points through Step 2 to Step 4.
Step 6: Compare (13) to the exposure threshold to decide whether each estimated scattering center becomes the main scattering center.

### 2.3. Computational Complexity

In this section, the computational complexity of the proposed algorithm is calculated and compared to that of the conventional methods. For comparison under the same conditions, only the process after obtaining HRRP through *S* frequency samples received through *N* observations was considered.

The proposed algorithm selects *K* out of *N* HRRPs to construct **h** in (7). Since the pseudo-inverse operation **P^†^** can be pre-calculated with the knowledge of observation angles, it is excluded from the complexity derivation. Considering the matrix multiplication for *Q* combinations of HRRPs, the complexity can be express as O(2KQ) using the Big-O notation. The error function in (9) uses *Q* scattering centers to estimate their authenticities; it also has the complexity of O(2KQ). For the actual target model, this process repeats approximately N times for estimating total scattering centers, which is assumed to be Mavg×N. Finally, the estimated total scattering centers are used in (10) to calculate the HRRPs for *N* observation angles in order to determine their dominance.

Therefore, the complexity becomes

(14) $$C_{prop}\approx O\left(4KQN+2M_{avg}N^{2}\right),$$

And with approximated *Q* as $M_{avg}^{K}$,

(15) $$C_{prop}\approx O\left(4KM_{avg}^{K}N+2M_{avg}N^{2}\right)$$

The computational complexity of the conventional SAR image algorithm can be expressed as the following equation [31,32]:

(16) $$C_{SAR}\approx O\left(S^{2}N\right)$$

For example, in the case of *K* = 4, *M_avg_* = 4, *N* = 121, and *S* = 1024, the complexities of the proposed method and SAR algorithm are $O(6.13\times 10^{5})$ and $O(1.27\times 10^{8})$, respectively. It can be seen that the proposed method has a complexity of about 0.5% of that of the SAR algorithm.

In [22], MEM network optimization was used in the scattering center estimation, and the associated computational complexity can be expressed as

(17) $$C_{MEM}\approx O\left(z\left(J+L\log L\right)\right)$$

where *z* is the number of iterations of the MEM operation and *J* is the number of neurons over the entire network, which is defined as P×*_N_*C_2_. The number of modules *P* should be larger than the number of scattering points *M*, and *N* is the number of observations. Finally, *L* is the number of neurons in one module defined as *_N_*C_2_.

In the case of N=10, M=4, P=8, and z=300, the complexity of the MEM-based method is $O(1.59\times 10^{5})$, while the proposed method has the complexity of $O(4.18\times 10^{4})$ with K=4. Moreover, under the same assumption as in [22], the method for the point target model can be used for the comparison, which means the complexity of $O(4.10\times 10^{3})$ can be reached for the given environment. It can be seen that the proposed method has a complexity of 26.3% and 2.6% compared to the algorithm proposed in [22].

## 3. Results and Discussion

### 3.1. Simulation on Numerical Model

**Figure 6:**
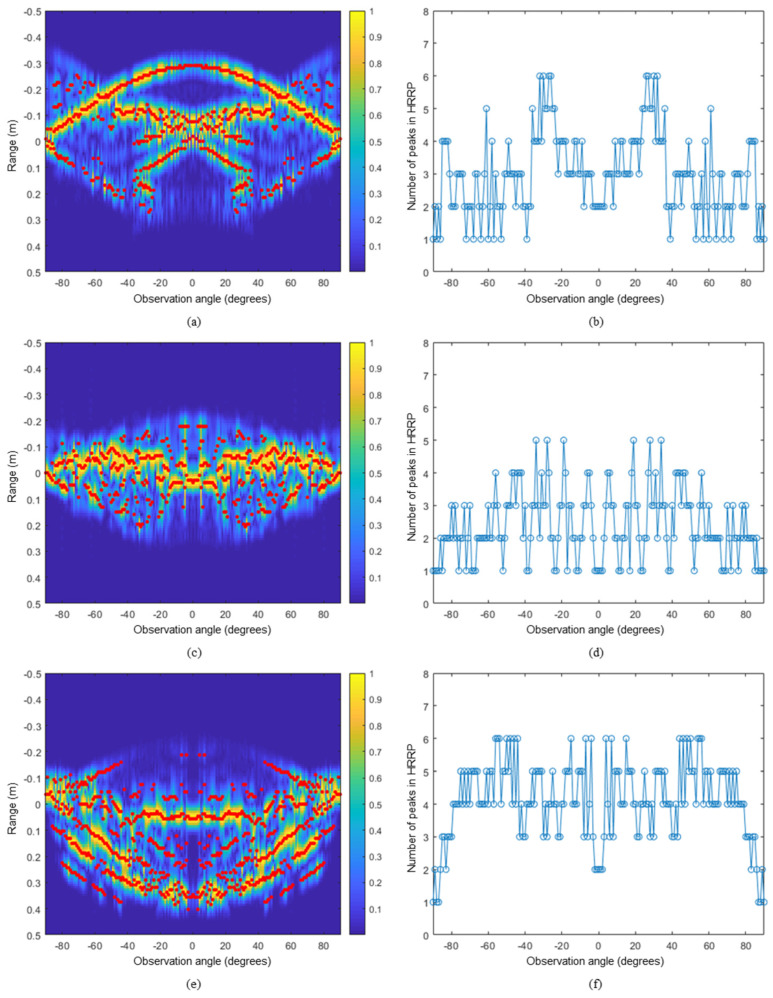
Peak detection for numerical models with the number of peaks in HRRPs with respect to the observation angle. A380: (**a**,**b**), Eurofighter: (**c**,**d**), F-15: (**e**,**f**).

**Figure 7:**
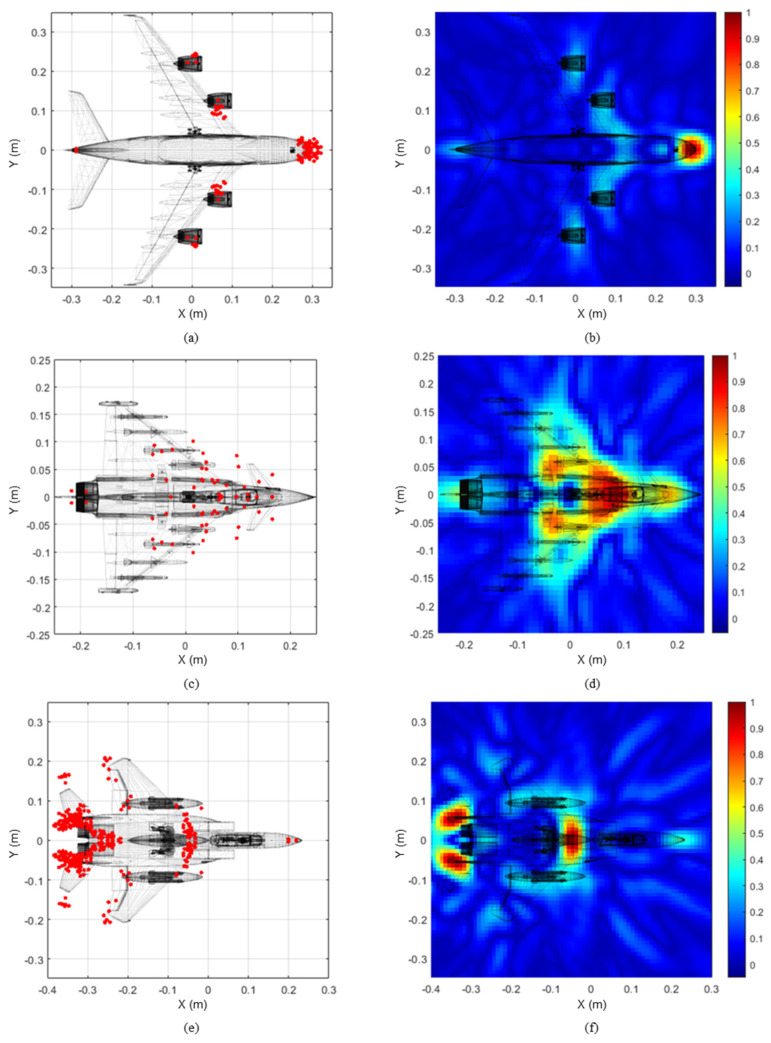
Two-dimensional scattering center estimation and corresponding SAR images of the A380: (**a**,**b**), Eurofighter: (**c**,**d**), and F-15: (**e**,**f**).

The proposed algorithm was applied to the reduced scale numerical models for three different types of aircraft: the airbus A380, the Eurofighter, and the F-15. The HRRP datasets were obtained from the CAD models at elevation angle θ=0° and azimuth angle ϕ in a range of −90° to 90° with an interval of 1° and observation angles *N* of 181 using the physical optics technique at the X-band, 8 GHz to 12 GHz. Furthermore, the material of the CAD model was assumed to be a PEC (Perfect Electric Conductor). The number of frequency steps, denoted as frequency samples *S*, was 1024, and the frequency step size was 3.9 MHz, with a range resolution Δ*R* of 3.75 cm. Moreover, a noise-free condition was assumed for effective comparison with the SAR technique. The specifications of the airborne targets and a list of the parameters used in the proposed algorithm are listed in Table 1 and Table 2, respectively. The proposed algorithm was applied to the HRRPs obtained for each numerical model, and the peak detection results are shown in Figure 6. The red dots in Figure 6a,c,e represent the detected peaks within the measured HRRPs for each observation angle. In addition, the number of peaks obtained for each observation angle is shown in Figure 6b,d,f, where the A380, Eurofighter, and F-15 had an average of 2.97, 2.38, and 4.1 HRRP peaks, respectively, regarding all observation angles. The different number of peaks confirms that the actual target model has different visible scattering points depending on the observation angle. The red dots in Figure 7a,c,e represent the estimated scattering centers in two-dimensional space using the angular HRRP peaks detected in Figure 6. The SAR images for corresponding models are shown in Figure 7b,d,f as well. From the results of SAR imaging shown in Figure 7, we can see that the scattering centers of the A380 arise at the nose, the jet engines, and the tail wing. The Eurofighter model has the scattering centers at its canopy, intake, and tail wing, while the F-15 model has the scattering centers at the intake, missiles, wing edges, and tail wing. We can see that the scattering centers estimated by the proposed algorithm are distributed similarly to those of the SAR images.

Finally, after 1000 trials on an Intel Core i7-4930 CPU @ 3.4 GHz, the average computation time of the proposed algorithm was 2.2 ms, which is only 2% of the 150 ms required for the SAR algorithm.

### 3.2. Experiment on Actual Target

To validate the proposed algorithm, a radar measurement system was designed consisting of Agilent’s 8720ES vector network analyzer (VNA) and two X-band standard gain horn antennas. The target is placed on a rotating platform for angular variation.

**Figure 8:**
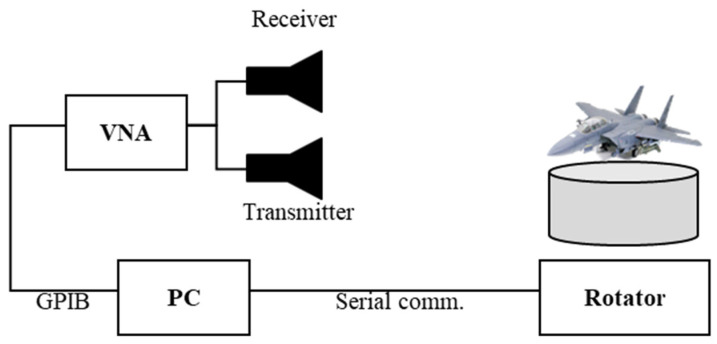
Block diagram of the entire measurement system.

**Figure 9:**
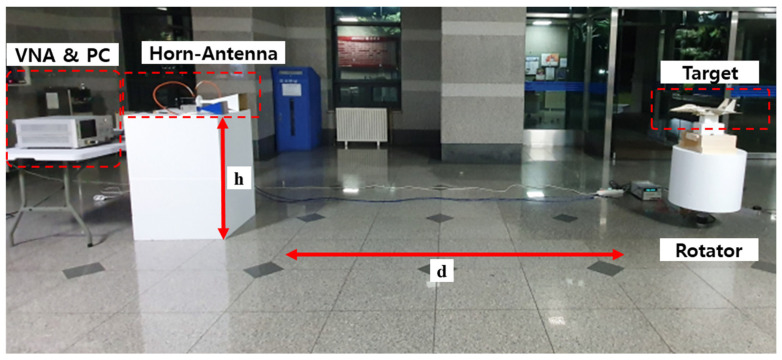
Experimental environment with d = 4.2 m and h = 1 m.

The VNA was used at the X-band, 8 GHz to 12 GHz, with 1024 frequency steps, denoted as frequency samples *S*, and the frequency step size was 3.9 MHz with a range resolution Δ*R* of 3.75 cm. Also, the desired transmit power is 5 dBm. The gain of the horn antenna was 20 dBi, and the measured data from the VNA was sent to a storage system via Agilent’s 82357B GPIB connection, which is manufactured by Keysight Technologies, located in Santa Clara, CA, USA. The azimuth and elevation angles of the rotating platform were controlled by an Autonics A200K-M599-G10 stepper motor which is manufactured by Autonics, located in Busan, South Korea and a MG995 geared servomotor which is manufactured by TowerPro, located in Taiwan, respectively, through serial communication. A block diagram of the entire radar measurement system is shown in Figure 8, and the experimental setup is shown in Figure 9.

As performed in the numerical simulation, the scattering centers of the plastic die-cast models of the airbus A-380, Eurofighter, and F-15 were estimated by applying the proposed algorithm. A conductive silver paste was applied to the plastic target surface to improve the signal response. Since the dimensions of each numerical model were designed to be the same as the die-cast model, they follow the specifications listed in Table 1.

The HRRP datasets were obtained for each model at an elevation of θ=0°, and azimuth angle ϕ in a range of −60° to 60° with an interval of 1° using the proposed measurement system, and the number of observation angles *N* was 121. The antenna was positioned at a height of 1 m above the ground at a distance of 4.2 m from the target. The same set of algorithm parameters was used as in the numerical simulation and is listed in Table 2.

**Figure 10:**
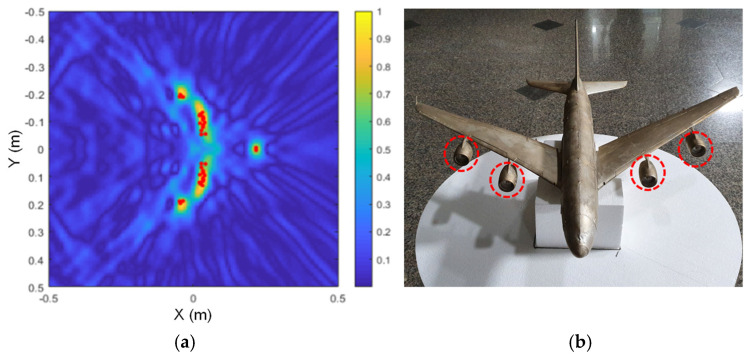
Estimated scattering centers of the A380 model; (**a**) drawn on a SAR image, and (**b**) the corresponding dominant scattering centers.

**Figure 11:**
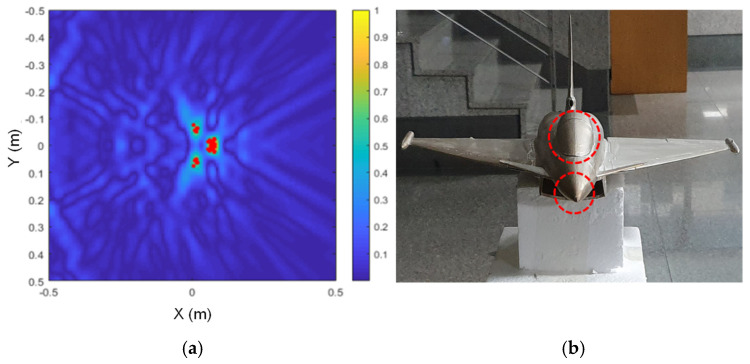
Estimated scattering centers of the Eurofighter model; (**a**) drawn on a SAR image, and (**b**) the corresponding dominant scattering centers.

**Figure 12:**
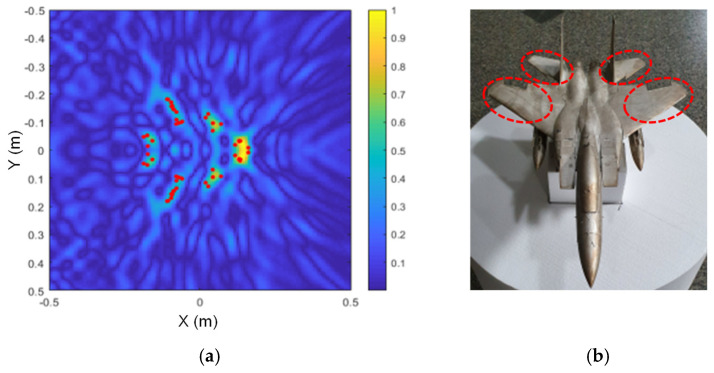
Estimated scattering centers of the F-15 model; (**a**) drawn on a SAR image, and (**b**) the corresponding dominant scattering centers.

The scattering point estimation results are shown in Figure 10, Figure 11 and Figure 12 as red dots on the SAR image obtained for each model, and the corresponding dominant scattering centers of the actual target are highlighted in red circles. As we have already seen from the simulation results, the dominant scattering centers are distributed at the main points of the SAR images, such as the engines, intakes, and the wings.

### 3.3. Target Classification

The scattering points estimated by the proposed algorithm are subject to being expressed in the standardized data dimension to be applied to the classifier machine. In order to standardize the positions of the estimated scattering centers, a scattering center image needs to be generated. In this case, the position and value of each pixel are required to consider the two-dimensional location of the scattering centers. Therefore, when the estimated scattering centers exist within the predefined two-dimensional pixel, it is determined that the image pixel exists, and the value of each pixel is defined as the maximum value of the amplitude of the scattering points within the pixel, which is defined as the sum of the amplitudes of *K* HRRPs used for scattering center estimation. In the classification process, the SVM is used, which has the advantage of classifying feature points that are difficult to linearly classify through high-dimensional mapping using a kernel function.

The HRRP datasets for each model were obtained at elevation θ and azimuth angles ϕ in ranges of −30° to 30° and −60° to 60°, respectively, with intervals of 2° and 1°, under the same conditions as in Experiment 3.2. The image was generated using HRRP in the azimuth section defined at a specific elevation angle, and the same pixel size and image size were considered for comparison of its classification performance to the SAR images. The size of the image used for training and validation is −0.5 m × 0.5 m, where the size of one pixel is 0.05 m × 0.05 m, so it has 21 pixels in width and depth respectively.

**Figure 13:**
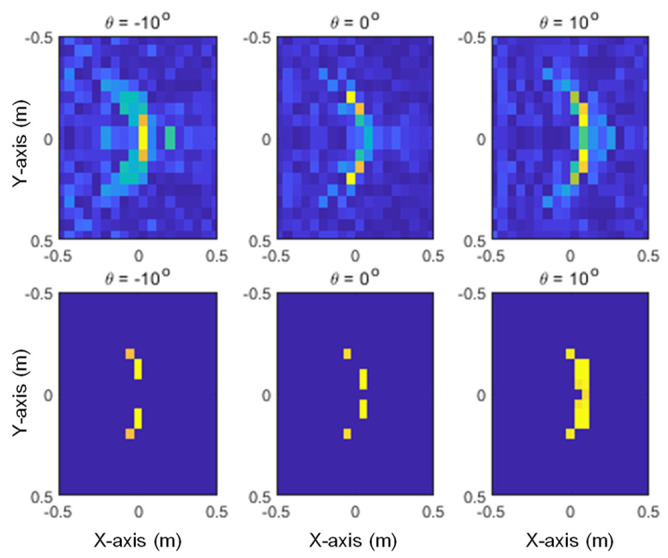
Generated images for the A380: SAR (**upper row**) and the proposed method (**lower row**).

**Figure 14:**
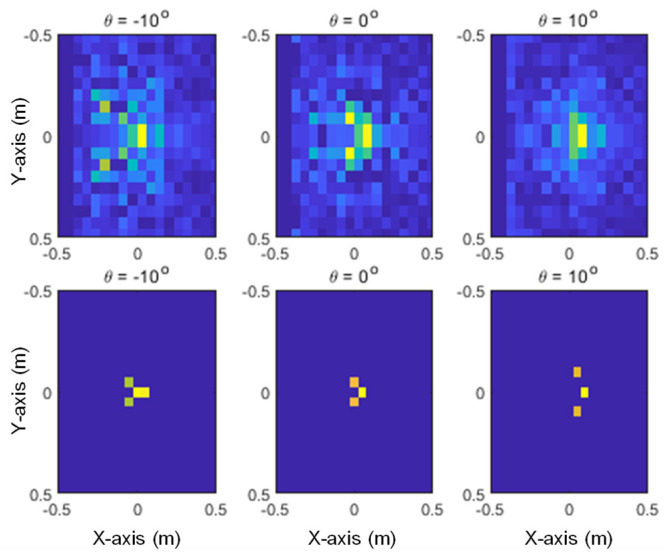
Generated images for the Eurofighter: SAR (**upper row**) and the proposed method (**lower row**).

**Figure 15:**
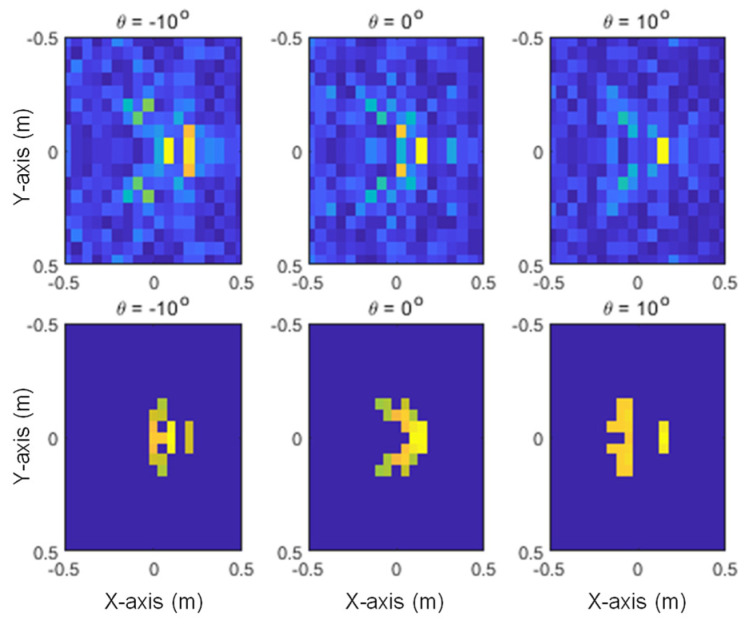
Generated images for the F-15: SAR (**upper row**) and the proposed method (**lower row**).

The images generated from the proposed method and SAR with three different elevation angles are shown in Figure 13, Figure 14 and Figure 15 for the given target models. In these figures, it can be seen that the pixels of the scattering center image are distributed at the main points of the SAR image, and it can also be confirmed that the characteristics of each target are clearly distinguishable.

The verification was performed by dividing the training set and validation set, as shown in Table 3. The azimuth section for image generation was defined as three sections, and the elevation section was defined to be different so that the images for training and verification did not overlap. For training and validation, a total of 31 images were generated for each model, where 16 images were used for training and 15 images were applied for validation.

To train and validate the classifier, feature extraction was performed using PCA (Principal Component Analysis) and LDA (Linear Discriminant Analysis). PCA reduces the dimensionality of high-dimensional input data by projecting it onto the dimensions with maximum variance. LDA, on the other hand, considers the data classes and finds a coordinate space that minimizes the variance within classes while maximizing the variance between classes. Using these techniques, distinctive feature points were extracted.

The feature points obtained from the training images were used to train an SVM (Support Vector Machine) with an RBF kernel. The feature points extracted from the validation images were then input into the trained SVM for classification.

**Figure 16:**
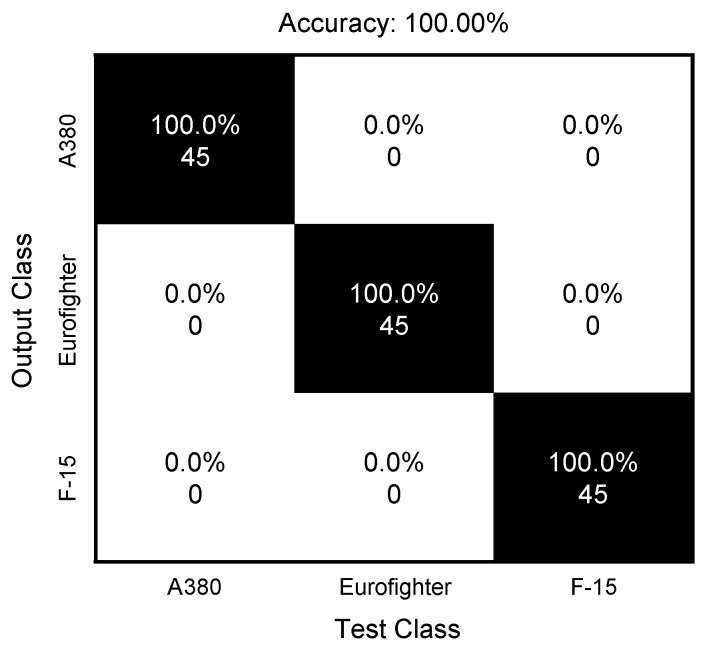
Classification results using SAR images.

**Figure 17:**
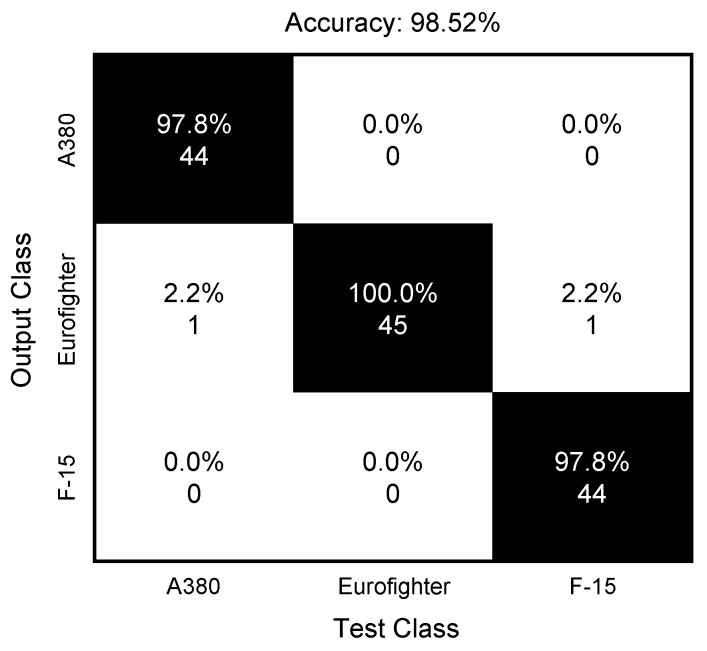
Classification results using the proposed method.

The classification results using the datasets described in Table 3 are shown as the confusion matrix form in Figure 16 and Figure 17. In the case of using a SAR image, it can be confirmed that all targets were classified in the right place, while the classification rate of the proposed algorithm is 98.52%, which is somewhat inferior to that of the SAR images. This can be seen as a performance loss due to the trade-off for reducing the computation load confirmed in the previous section. Based on the results, it can be judged that the proposed algorithm can be applied to situations requiring real-time target recognition or two-dimensional target information.

## 4. Conclusions

In this paper, we proposed a new method for estimating target scattering centers from multiple HRRP datasets. These scattering centers can help obtain a high resolution image of the target and thus can also be used for the classification of the target. Based on the range information contained in multiple HRRPs obtained from various observation angles, the estimated target scattering centers could be located at the intersection points of the lines perpendicular to each of the observation axes. The computational complexity of the proposed algorithm was proven to be significantly less than that of the conventional SAR method or other methods in the previous literature.

The performance of the proposed algorithm was verified by applying the multiple HRRPs measured in the experiment using the X-band radar measurement system. The system was designed using the VNA, a standard gain horn antenna, and a rotator for angle rotation, and the HRRP dataset was obtained using the received signal. As a result of estimating the scattering centers by applying the proposed algorithm to the obtained HRRPs, it was confirmed that the scattering centers can be successfully estimated at the main points of the SAR images.

Finally, a strategy for generating images was introduced to be used in the classifier from the estimated scattering centers. The classification performance using the scattering center image had a failure rate of less than 2% compared to the discrimination performance using the SAR image.

As demonstrated in this article, the proposed algorithm shows similar target classification capabilities but much less necessary computation than the SAR-based method. Therefore, the proposed algorithm is advantageous in real-time image acquisition and the reduction of the required storage capacity of the classification database, so it is expected to be used in various fields, from defense fields, such as air defense, maritime, and ground surveillance, to private industries, such as vehicle radar and drone radar.

## Figures and Tables

**Table 1:** Specifications of target models.

Model (Scale)	Length (mm)	Wingspan (mm)
A380 (1:120)	650	580
Eurofighter (1:32)	500	350
F-15 (1:32)	600	400

**Table 2:** Algorithm parameters for numerical evaluation.

Parameter	Value
Number of angle selections K	4
Angle interval δ	3°
Matching threshold γ	Δ*R*/2
Exposure threshold γ_ρ_	70%

**Table 3:** HRRP acquisition angle sections for training and testing.

Item	Azimuth (deg)	Step	Elevation (deg)	Step
Training	−40°~40°	1°	−30°~30°	4°
	−50°~50°			
	−60°~60°			
Validation	−40°~40°	1°	−28°~28°	4°
	−50°~50°			
	−60°~60°			

## Data Availability

The data are contained within the article.
